# Supplementary material for: Generation of transgenic mice expressing a FRET biosensor, SMART, that responds to necroptosis
Source: Commun Biol. 2022 Dec 5;5:1331. doi: 10.1038/s42003-022-04300-0 (PMC9722793; doi:10.1038/s42003-022-04300-0)
Supplement: Supplementary file 2 — Supplementary Information [file 42003_2022_4300_MOESM2_ESM.pdf]

Supplementary Information

Generation of transgenic mice expressing a FRET biosensor, SMART, that responds to necroptosis

Shin Murai[1], Kanako Takakura[2], Kenta Sumiyama[3], Kenta Moriwaki[1], Kenta Terai[4], Sachiko Komazawa-Sakon[1], Takao Seki[1], Yoshifumi Yamaguchi[5], Tetuo Mikami[6], Kimi Araki[7,8], Masaki Ohmuraya[9], Michiyuki Matsuda[4, 10, 11], Hiroyasu Nakano

Supplementary Figures 1-3

Supplementary Movies 1 - 6

Supplementary Movie 1. Imaging of necroptosis in peritoneal macrophages, Related to Figure 1.
Peritoneal macrophages from SMART Tg mice were stimulated with BV6/zVAD.

Supplementary Movie 2. Imaging of pyroptosis in peritoneal macrophages, Related to Figure 2.
Peritoneal macrophages from SMART Tg mice were primed with LPS, then stimulated with nigericin.

Supplementary Movie 3. Imaging of necroptosis in MEFs, Related to Figure 4.
MEFs from SMART Tg mice were stimulated with TNF/BV6/zVAD.

Supplementary Movie 4. Imaging of intestinal epithelial cells of the ileum of SMART

Tg mice, Related to Supplementary Figure 3.
SMART Tg mice were injected with mTNF, and the ileum was analyzed by two-photon excitation microscopy. The time (min) indicates the time after TNF injection.

Supplementary Movie 5. Imaging of the renal proximal tubular cells in the kidney of untreated SMART Tg mice, Related to Figure 6.
SMART Tg mice were untreated, and the kidney was analyzed by two-photon excitation microscopy. The time (min) since the movie started on day 2 is shown.

Supplementary Movie 6. Imaging of the renal proximal tubular cells in the kidney of CDDP-treated SMART Tg mice, Related to Figure 6.
SMART Tg mice were treated with CDDP, and then the kidney was analyzed by two-photon excitation microscopy on day 2 following CDDP injection. The time (min) since the movie started on day 2 is shown.

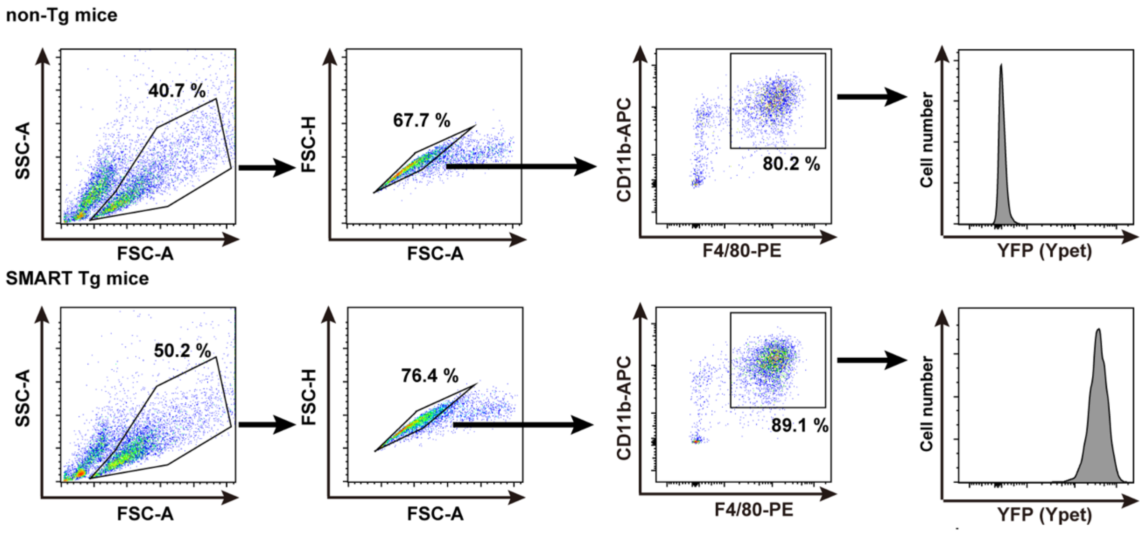

Supplementary Figure 1. Gating strategy. Peritoneal cells were isolated from 8- to 12-week-old non-Tg or SMART Tg mice were analyzed by flow cytometer.

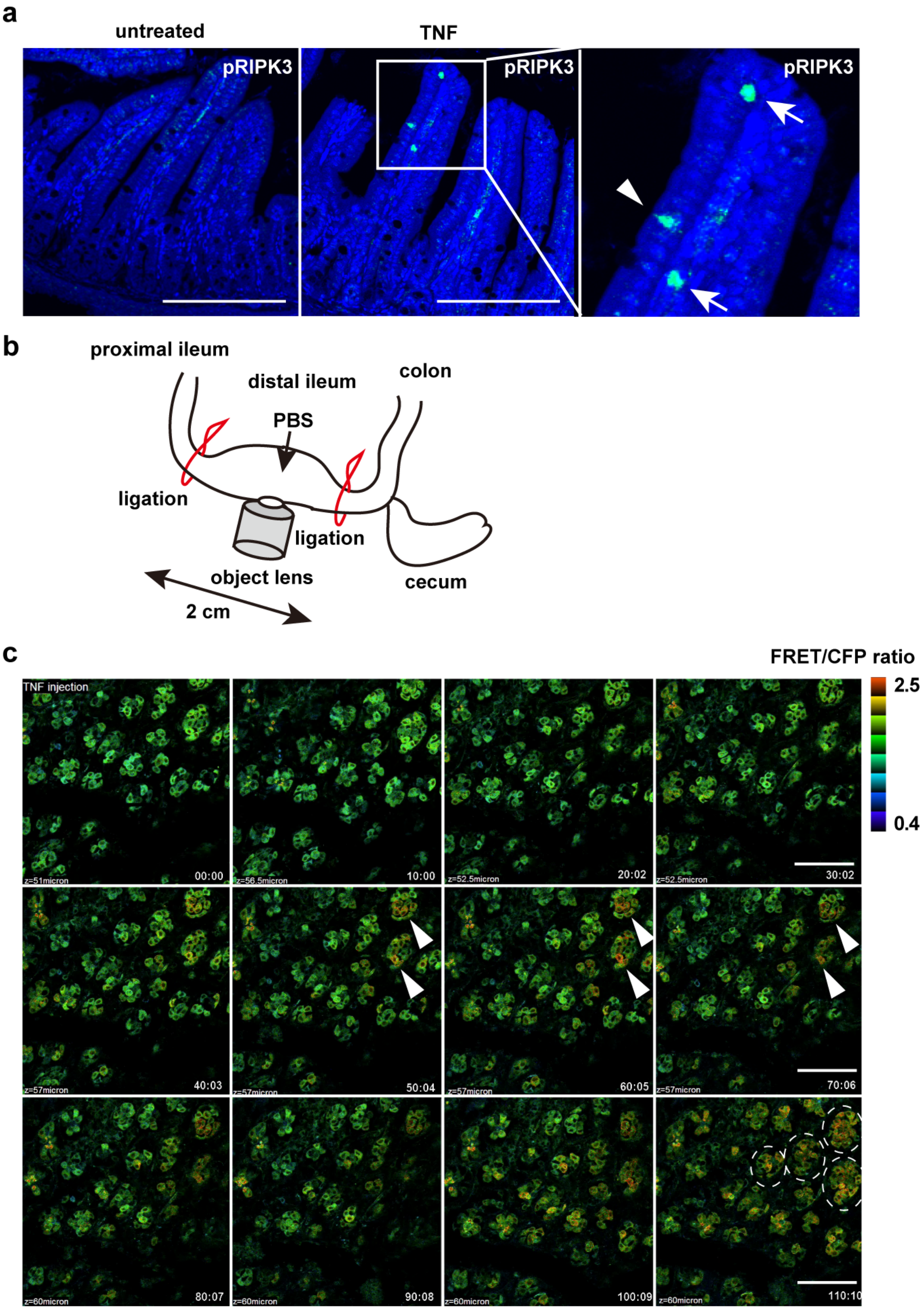
a
untreated
TNF
pRIPK3
pRIPK3
pRIPK3
b
proximal ileum
distal ileum
colon
PBS
ligation
ligation
object lens
cecum
2 cm
c
FRET/CFP ratio
2.5
0.4
TNF injection
00:00
10:00
20:02
30:02
40:03
50:04
60:05
70:06
80:07
90:08
100:09
110:10

Supplementary Figure 2. Imaging of the ileum of TNF-injected mice by two-photon excitation microscopy. **a**, Eight-week-old wild-type mice were intravenously injected with mTNF (0.5 mg/kg) and mice were sacrificed at 3 hours after TNF injection. Ileal tissue sections were stained with phospho-specific RIPK3 (pRIPK3) antibody (n = 3 mice). The right side is an enlarged image of the white box in the middle. White arrowhead and arrows indicate pRIPK3^{+} epithelial cells and pRIPK3^{+} non-epithelial cells, respectively. Scale bar, 100 μm. **b**, Procedure of imaging of the ileum by two-photon excitation microscopy. After incision of the median line of the abdominal wall of 8- to 12-week-old SMART Tg mice, the ileum was pulled out of the abdominal cavity. To reduce peristalsis, 2 cm long ileum was loosely ligated at rostral and caudal sides and then filled with PBS. Then, mice were intravenously injected with mTNF (0.5 mg/kg) and analyzed by two-photon excitation microscopy. **c**, Pseudocolored images of the FRET/CFP ratios in the ileum of TNF-injected mice. FRET/CFP responses are color-coded according to the color scales. Scale bars, 100 μm. Times indicate times after TNF injection. White arrowheads indicate cells that show an increase in the FRET/CFP ratio. Dotted white circles indicate cells showing an increase in the FRET/CFP ratio simultaneously, possibly due to ischemia.

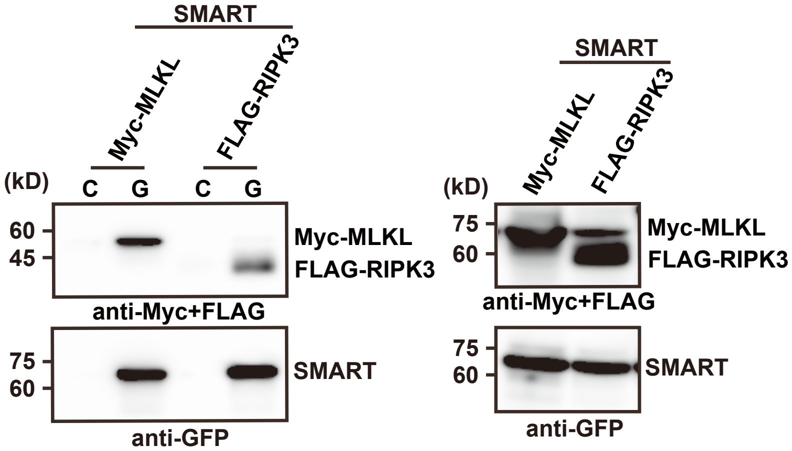

Supplementary Figure 3. SMART interacts with MLKL and RIPK3. HEK293 cells were transiently transfected with expression vectors for SMART along with Myc-tagged MLKL or FLAG-tagged RIPK3. After 24 hours, cell were lysed and immunoprecipitated with GST (Control, C) or GST-fused anti-GFP nanobody (G) adsorbed to Glutathione-Sepharose 4B beads. Immunoprecipitated proteins were analyzed by anti-Myc and anti-FLAG antibodies, and then reblotted with anti-GFP antibody. Expression of transfected proteins was verified using total cell lysates by immunoblotting with the indicated antibodies. Results are representative of two independent experiments.
