# Supplementary material for: Generation of transgenic mice expressing a FRET biosensor, SMART, that responds to necroptosis
Source: Commun Biol. 2022 Dec 5;5:1331. doi: 10.1038/s42003-022-04300-0 (PMC9722793; doi:10.1038/s42003-022-04300-0)
Supplement: Supplementary file 3 — Description of Additional Supplementary Data [file 42003_2022_4300_MOESM3_ESM.pdf]

Description of Additional Supplementary Files

File name: Supplementary Data

Description: The source data behind the graphs in the paper

File name: Supplementary Movie 1

Description: Imaging of necroptosis in peritoneal macrophages

File name: Supplementary Movie 2

Description: Imaging of pyroptosis in peritoneal macrophages

File name: Supplementary Movie 3

Description: Imaging of necroptosis in MEFs

File name: Supplementary Movie 4

Description: Imaging of intestinal epithelial cells of the ileum of SMART Tg mice

File name: Supplementary Movie 5

Description: Imaging of the renal proximal tubular cells in the kidney of untreated SMART Tg mice

File name: Supplementary Movie 6

Description: Imaging of the renal proximal tubular cells in the kidney of CDDP-treated SMART Tg mice
