# Supplementary material for: Generation of transgenic mice expressing a FRET biosensor, SMART, that responds to necroptosis
Source: Commun Biol. 2022 Dec 5;5:1331. doi: 10.1038/s42003-022-04300-0 (PMC9722793; doi:10.1038/s42003-022-04300-0)
Supplement: Supplementary file 11 — nr-reporting-summary [file 42003_2022_4300_MOESM11_ESM.pdf]

nature portfolio

Corresponding author(s): Hiroyasu Nakano

Last updated by author(s): November 12, 2022

Reporting Summary

Nature Portfolio wishes to improve the reproducibility of the work that we publish. This form provides structure for consistency and transparency in reporting. For further information on Nature Portfolio policies, see our Editorial Policies and the Editorial Policy Checklist.

Statistics

For all statistical analyses, confirm that the following items are present in the figure legend, table legend, main text, or Methods section.

n/a	Confirmed	
☐	☒	The exact sample size (*n*) for each experimental group/condition, given as a discrete number and unit of measurement
☐	☒	A statement on whether measurements were taken from distinct samples or whether the same sample was measured repeatedly
☐	☒	The statistical test(s) used AND whether they are one- or two-sided<br>*Only common tests should be described solely by name; describe more complex techniques in the Methods section.*
☐	☒	A description of all covariates tested
☐	☒	A description of any assumptions or corrections, such as tests of normality and adjustment for multiple comparisons
☐	☒	A full description of the statistical parameters including central tendency (e.g. means) or other basic estimates (e.g. regression coefficient) AND variation (e.g. standard deviation) or associated estimates of uncertainty (e.g. confidence intervals)
☐	☒	For null hypothesis testing, the test statistic (e.g. *F*, *t*, *r*) with confidence intervals, effect sizes, degrees of freedom and *P* value noted<br>*Give P values as exact values whenever suitable.*
☒	☐	For Bayesian analysis, information on the choice of priors and Markov chain Monte Carlo settings
☒	☐	For hierarchical and complex designs, identification of the appropriate level for tests and full reporting of outcomes
☒	☐	Estimates of effect sizes (e.g. Cohen's *d*, Pearson's *r*), indicating how they were calculated

Our web collection on statistics for biologists contains articles on many of the points above.

Software and code

Policy information about availability of computer code

Data collection	BD FACSDiva (BD Bioscience), Amersham Imager 600 (GE Healthcare Life Sciences), SoftWoRx (Applied Precision Inc.), MetaMorph (Molecular devices), ZEN 2 software (ZEISS), BZ-X Viewer (Keyence)
Data analysis	FlowJo 10.6 (BD Bioscience), MetaMorph (Molecular devices), ImageJ2,ver2.3 (NIH), Graph Pad Prism 9, Micorsoft Excel, BZ-X Analyzer (Keyence),

For manuscripts utilizing custom algorithms or software that are central to the research but not yet described in published literature, software must be made available to editors and reviewers. We strongly encourage code deposition in a community repository (e.g. GitHub). See the Nature Portfolio guidelines for submitting code & software for further information.

Data

Policy information about availability of data

All manuscripts must include a data availability statement. This statement should provide the following information, where applicable:

- Accession codes, unique identifiers, or web links for publicly available datasets
- A description of any restrictions on data availability
- For clinical datasets or third party data, please ensure that the statement adheres to our policy

The data supporting of this study are available within the paper and its supplementary movies. Source data behind the graphs available as source data files. Other datasets generated during and/or analyzed during the current study are available from the corresponding author upon reasonable request.

Human research participants

Policy information about studies involving human research participants and Sex and Gender in Research.

Reporting on sex and gender	*Use the terms sex (biological attribute) and gender (shaped by social and cultural circumstances) carefully in order to avoid confusing both terms. Indicate if findings apply to only one sex or gender; describe whether sex and gender were considered in study design whether sex and/or gender was determined based on self-reporting or assigned and methods used. Provide in the source data disaggregated sex and gender data where this information has been collected, and consent has been obtained for sharing of individual-level data; provide overall numbers in this Reporting Summary. Please state if this information has not been collected. Report sex- and gender-based analyses where performed, justify reasons for lack of sex- and gender-based analysis.*
Population characteristics	*Describe the covariate-relevant population characteristics of the human research participants (e.g. age, genotypic information, past and current diagnosis and treatment categories). If you filled out the behavioural & social sciences study design questions and have nothing to add here, write "See above."*
Recruitment	*Describe how participants were recruited. Outline any potential self-selection bias or other biases that may be present and how these are likely to impact results.*
Ethics oversight	*Identify the organization(s) that approved the study protocol.*

Note that full information on the approval of the study protocol must also be provided in the manuscript.

Field-specific reporting

Please select the one below that is the best fit for your research. If you are not sure, read the appropriate sections before making your selection.

☒ Life sciences ☐ Behavioural & social sciences ☐ Ecological, evolutionary & environmental sciences

For a reference copy of the document with all sections, see nature.com/documents/nr-reporting-summary-flat.pdf

Life sciences study design

All studies must disclose on these points even when the disclosure is negative.

Sample size	Sample size calculations were not performed. At least 2 or 3 independent experiments were performed both cultured cells and mice.
Data exclusions	No data were excluded from the analysis.
Replication	For all experiments, 2 or 5 experiments were performed.
Randomization	Samples were allocated to experimental group without any previous selection.
Blinding	For the experiments which involved treating primary cells with different stimuli/inhibitors, blinding was not possible with this experimental design. Moreover, blinding was not performed for the quantitation of immunofluorescent signals as these analyses used unbiased macros which quantified all data that had been captured for each experiment. For the animal experiments, each genotypes of mice were not blinding because it was unsuitable for the comparison of results among mice, but the analyzing parameters were identical among the experiments

Reporting for specific materials, systems and methods

We require information from authors about some types of materials, experimental systems and methods used in many studies. Here, indicate whether each material, system or method listed is relevant to your study. If you are not sure if a list item applies to your research, read the appropriate section before selecting a response.

Materials & experimental systems

n/a	Involved in the study	
☐	☒	Antibodies
☒	☐	Eukaryotic cell lines
☒	☐	Palaeontology and archaeology
☐	☒	Animals and other organisms
☒	☐	Clinical data
☒	☐	Dual use research of concern

Methods

n/a	Involved in the study	
☒	☐	ChIP-seq
☐	☒	Flow cytometry
☒	☐	MRI-based neuroimaging

March 2021

Antibodies

Antibodies used	anti-actin (A2066, Sigma-Aldrich)<br>anti-cleaved caspase 3 (9661, Cell Signaling)<br>APC–conjugated anti-CD11b (20-0112, TOMBO Biosciences)<br>PE–conjugated anti-F4/80 (50-4801, TOMBO biosciences)<br>anti-FLAG (M2, F3165, Sigma-Aldrich)<br>anti-GFP (sc-8334, Santa Cruz)<br>anti-Myc (9E10, 05419, Sigma-Aldrich)<br>anti-MLKL (3H1, Millipore)<br>anti-phospho-RIPK3 (57220, Cell Signaling)<br>horseradish peroxidase (HRP)–conjugated sheep anti-mouse IgG (NA931, GE Healthcare)<br>HRP–conjugated donkey anti-rat IgG (712-035-153, Jackson ImmunoResearch)<br>HRP–conjugated donkey anti-rabbit IgG (NA934, GE Healthcare)<br>biotin-conjugated goat anti-rabbit IgG (E0432, Dako)<br>GST-fused anti-GFP nanobody (61838, Addgene)
Validation	All antibodies that came from commercial vendors are validated by the manufactures for the species and assays in our study. Validation data is available on the manufacture's websites.<br><br>anti-actin: https://www.sigmaaldrich.com/JP/ja/product/sigma/a2066<br>anti-cleaved caspase 3: https://www.cellsignal.jp/products/primary-antibodies/cleaved-caspase-3-asp175-antibody/9661<br>APC–conjugated anti-CD11b: https://tonbobio.com/products/apc-anti-human-mouse-cd11b-m1-70<br>PE–conjugated anti-F4/80: https://tonbobio.com/products/pe-anti-mouse-f4-80-antigen-bm8-1<br>anti-FLAG: https://www.sigmaaldrich.com/JP/ja/product/sigma/f3165<br>anti-GFP: https://www.scbt.com/p/gfp-antibody-fl?productCanUrl=gfp-antibody-fl&_requestid=1774250<br>anti-MLKL: https://www.merckmillipore.com/JP/ja/product/Anti-MLKL-Antibody-clone-3H1,MM_NF-MABC604<br>anti-Myc: https://www.sigmaaldrich.com/JP/ja/product/mm/05419<br>anti-phospho-RIPK3: https://www.cellsignal.com/products/primary-antibodies/phospho-rip3-thr231-ser232-antibody-mouse-specific/57220<br>GST-fused anti-GFP nanobody: https://www.addgene.org/61838/<br>GST-fused anti-GFP nanobody was purified in house as a standard procedure. The antibody was validated by immunoprecipitation using cell lysates transfected with the expression vectors for GFP-tagged proteins.

Animals and other research organisms

Policy information about studies involving animals; ARRIVE guidelines recommended for reporting animal research, and Sex and Gender in Research

Laboratory animals	All mouse strains are described in the material and methods section. The age of the mice used in each experiment is either stated in the methods section and/or in the figure legends for each experiment.<br><br>The mice were housed at 23 ± 2°C, a humidity of 55 ± 5%, and a 12 hr dark/light cycle.
Wild animals	The study did not use wild animals.
Reporting on sex	Both female and male mice were involved.
Field-collected samples	The study did not involve field-collected samples.
Ethics oversight	All animal experiments were performed according to the guidelines approved by the Institutional Animal Experiments Committee of Toho University School of Medicine (approved number: 21-54-400), Kyoto Graduate School of Medicine (approved number: Medkyo 21562), and the RIKEN Kobe branch (approved number: QA2013-04-10).

Note that full information on the approval of the study protocol must also be provided in the manuscript.

Flow Cytometry

Plots

Confirm that:

☒ The axis labels state the marker and fluorochrome used (e.g. CD4-FITC).

☒ The axis scales are clearly visible. Include numbers along axes only for bottom left plot of group (a 'group' is an analysis of identical markers).

☒ All plots are contour plots with outliers or pseudocolor plots.

☒ A numerical value for number of cells or percentage (with statistics) is provided.

Methodology

Sample preparation	For the isolation of peritoneal exudate cells (PEC)-macrophages, we intraperitoneally injected 6- to 8-week-old mice of the indicated genotype with 2.5 ml of 3% thioglycollate. On day 4 after thioglycollate injection, anesthetized mice were intraperitoneally injected and washed with ice-cold PBS, and then PBS was recovered. The same procedure was repeated twice. After removing non-adherent cells, cells were used as peritoneal macrophages.
Instrument	FACSCanto™ II Flow Cytometer (BD Bioscience)
Software	FlowJo™ 10.6 (BD Bioscience)
Cell population abundance	In all the experiments, unstained samples were used as control samples and at least 10,000 events were acquired. At least 80% of prepared peritoneal exudate cells were PEC-macrophages. Purity of the population was assessed with a CD11b and F4/80 antibodies.
Gating strategy	At first, the forward and side scatter parameters (FSC, SSC) gates were applied to exclude cell debris and doublets. The FSC/SSC gating samples were stained with CD11b and F4/80 antibodies and then gate for CD11b and F4/80 to define PEC-macrophages from all living cells. The single stained samples with CD11b or F4/80 was used to determine the boundaries between positive and negative staining.

☒ Tick this box to confirm that a figure exemplifying the gating strategy is provided in the Supplementary Information.
